# Perceptions of Family Caregivers of People With Dementia Regarding Symptom Management and the COVID-19 Pandemic

**DOI:** 10.1093/geroni/igab046.587

**Published:** 2021-12-17

**Authors:** Melissa Harris, Marita Titler

**Affiliations:** 1 Duke University, Durham, North Carolina, United States; 2 University of Michigan, Ann Arbor, Michigan, United States

## Abstract

Nearly 98% of older adults with Alzheimer’s disease and related dementias (ADRD) experience behavioral and psychological symptoms of dementia (BPSD). Although BPSD are linked to caregiver burden, perceptions of family caregivers on the impact of BPSD and their experiences addressing them in the home are unclear, and little is known about the impact of the pandemic on these experiences. Study aims were to explore: 1) the experiences of family caregivers of community dwelling older adults with ADRD regarding BPSD and how they manage BPSD in the home, and 2) how the pandemic impacted family caregivers’ experiences, BPSD of their relatives, and BPSD management. A qualitative, exploratory approach was used; 21 family caregivers were interviewed virtually. Content analysis and constant comparative methods were used. Ten major themes emerged: 1) Emotional and psychological responses of caregiver, 2) Loss, 3) Anticipation, 4) Reliance, 5) Learning to caregive, 6) Rewarding, 7) Emotional and psychological responses of care recipient 8) Cognition of care recipient, 9) Care strategies, 10) Caregiver perspectives. Caregivers did not use terms “behaviors” or “symptoms”, instead they described their relatives’ and their own experiences interdependently. Caregiving challenges presented before the pandemic (e.g. equivocal effects of medications, increasing care demands), many of which were compounded by the pandemic. Future research should explore the experiences of caregivers from a range of backgrounds. Findings illustrate communication barriers exist between clinicians, community services, people with ADRD and their families which may be addressed through clinician education, family-centered care planning, and policies to expand support service access.

